# Supplementary material for: Influence of social determinants of health on quality of life in patients with multimorbidity and polypharmacy
Source: PLoS One. 2024 Sep 26;19(9):e0297702. doi: 10.1371/journal.pone.0297702 (PMC11426528; doi:10.1371/journal.pone.0297702)
Supplement: S1 Table — (DOC) [file pone.0297702.s001.doc]

**Supplementary table 1.** List of chronic conditions analysed for the definition of multimorbidity based on the study by O’Halloran et al.1

| **CHAPTER** | **ICPC-2^2^** | **DESCRIPTION** |
| --- | --- | --- |
| **A** | A04 | General weakness/ tiredness |
|  | A70 | Tuberculosis |
|  | A79 | Malignancy, NOS^3^ |
|  | A90 | Congenital anomaly, NOS/ multiple |
| **B** | B72 | Hodgkin's disease/ lymphoma |
|  | B73 | Leukaemia |
|  | B74 | Malignant blood neoplasm, other |
|  | B75 | Benign/ unspecified blood neoplasm |
|  | B78 | Hereditary haemolytic anaemia |
|  | B81 | Anaemia, Vitamin B12/ folate deficiency |
|  | B82 | Anaemia, other/ unspecified |
|  | B83 | Purpura/ coagulation defect |
|  | B90 | HIV^4^ infection/ AIDS |
| **D** | D72 | Viral hepatitis |
|  | D74 | Malignant neoplasm, stomach |
|  | D75 | Malignant neoplasm, colon/ rectum |
|  | D76 | Malignant neoplasm, pancreas |
|  | D77 | Malignant digestive neoplasm, other/ NOS |
|  | D81 | Congenital anomaly of digestive system |
|  | D84 | Oesophagus disease |
|  | D85 | Duodenal ulcer |
|  | D86 | Peptic ulcer, other |
|  | D92 | Diverticular disease |
|  | D93 | Irritable bowel syndrome |
|  | D94 | Chronic enteritis/ ulcerative colitis |
|  | D97 | Liver disease, NOS |
|  | D98 | Cholecystitis/ cholelithiasis |
|  | D99 | Disease of digestive system, other |
| **F** | F74 | Neoplasm of eye/ adnexa |
|  | F83 | Retinopathy |
|  | F84 | Macular degeneration |
|  | F92 | Cataract |
|  | F93 | Glaucoma |
|  | F94 | Blindness |
| **H** | H75 | Neoplasm of ear |
|  | H82 | Vertiginous syndrome |
|  | H84 | Presbyacusis |
|  | H86 | Deafness |
| **K** | K71 | Rheumatic fever/ heart disease |
|  | K72 | Cardiovascular neoplasm |
|  | K73 | Cardiovascular congenital anomaly |
|  | K74 | Ischaemic heart disease with angina |
|  | K75 | Acute myocardial infarction |
|  | K76 | Ischaemic heart disease w/o angina |
|  | K77 | Heart failure |
|  | K78 | Atrial fibrillation/ flutter |
|  | K79 | Paroxysmal tachycardia |
|  | K80 | Cardiac arrhythmia, NOS |
|  | K81 | Heart/ arterial murmur, NOS |
|  | K82 | Pulmonary heart disease |
|  | K83 | Heart valve disease, NOS |
|  | K84 | Heart disease, other |
|  | K86 | Hypertension, uncomplicated |
|  | K87 | Hypertension, complicated |
|  | K88 | Postural hypotension |
|  | K89 | Transient cerebral ischaemia |
|  | K90 | Stroke/ cerebrovascular accident |
|  | K91 | Cerebrovascular disease |
|  | K92 | Atherosclerosis/ PVD^5^ |
|  | K93 | Pulmonary embolism |
|  | K94 | Phlebitis/ thrombophlebitis |
|  | K95 | Varicose veins of leg |
| **L** | L71 | Malignant musculoskeletal neoplasm |
|  | L82 | Congenital musculoskeletal anomaly |
|  | L83 | Neck syndrome |
|  | L84 | Back syndrome w/o radiating pain |
|  | L85 | Acquired deformity of spine |
|  | L86 | Back syndrome with radiating pain |
|  | L88 | Rheumatoid/ seropositive arthritis |
|  | L89 | Osteoarthrosis of hip |
|  | L90 | Osteoarthrosis of knee |
|  | L91 | Osteoarthrosis, other |
|  | L92 | Shoulder syndrome |
|  | L93 | Tennis elbow |
|  | L95 | Osteoporosis |
|  | L99 | Musculoskeletal disease, other |
| **N** | N73 | Neurological infection, other |
|  | N74 | Malignant neoplasm of nervous system |
|  | N75 | Benign neoplasm of nervous system |
|  | N76 | Neoplasm of nervous system, unspecific |
|  | N85 | Congenital abnormalities of the nervous system |
|  | N86 | Multiple sclerosis |
|  | N87 | Parkinson's disease/ parkinsonism |
|  | N88 | Epilepsy |
|  | N89 | Migraine |
|  | N90 | Cluster headache |
|  | N92 | Trigeminal neuralgia |
|  | N93 | Carpal tunnel syndrome |
|  | N94 | Peripheral neuritis/ neuropathies |
|  | N99 | Neurological diseases, other |
| **P** | P15 | Chronic alcohol abuse |
|  | P70 | Dementia |
|  | P71 | Organic psychosis, other |
|  | P72 | Schizophrenia |
|  | P73 | Affective psychosis |
|  | P74 | Anxiety state or disorder |
|  | P75 | Somatisation disorder |
|  | P76 | Depressive disorder |
|  | P78 | Neuraesthenia/ surmenage |
|  | P79 | Phobia/ compulsive disorder |
|  | P80 | Personality disorder |
|  | P8 | Hyperkinetic disorder |
|  | P82 | Post-traumatic stress disorder |
|  | P85 | Mental retardation |
|  | P86 | Anorexia nervosa/ bulimia |
|  | P98 | Other psychoses, NOS |
|  | P99 | Psychological disorders, other |
| **R** | R84 | Malignant neoplasm of trachea/bronchi/lung/pleura |
|  | R85 | Other malignant neoplasms of the respiratory system |
|  | R90 | Hypertrophy/ chronic infection of tonsils/ adenopathy |
|  | R95 | COPD^6^ |
|  | R96 | Asthma |
|  | R99 | Other respiratory system diseases |
| **S** | S77 | Malignant neoplasms of the skin |
|  | S86 | Seborrheic dermatitis |
|  | S87 | Atopic dermatitis/ eczema |
|  | S91 | Psoriasis |
|  | S96 | Acne |
|  | S99 | Other skin diseases |
| **T** | T71 | Malignant neoplasm of thyroid |
|  | T73 | Other endocrine/ unspecified neoplasias |
|  | T80 | Congenital endocrine/ metabolic abnormalities |
|  | T81 | Goiter |
|  | T82 | Obesity |
|  | T83 | Overweight |
|  | T85 | Hyperthyroidism/ thyrotoxicosis |
|  | T86 | Hypothyroidism/ Myxedema |
|  | T89 | Insulin-dependent diabetes |
|  | T90 | Non-insulin-dependent diabetes |
|  | T92 | Gout |
|  | T93 | Lipid metabolism disorders |
|  | T99 | Other endocrine/ metabolic/ nutritional problems |
| **U** | U75 | Malignant neoplasms of the kidney |
|  | U76 | Malignant neoplasms of the urinary bladder |
|  | U77 | Other malignant neoplasms of the urinary system |
|  | U88 | Nephrosis/ glomerulonephritis |
|  | U99 | Other urinary problems/ diseases |
| **W** | W15 | Female infertility/ subfertility |
|  | W72 | Malignant neoplasm related to fertility |
| **X** | X74 | Pelvic inflammatory disease |
|  | X75 | Malignant neoplasms of the cervix |
|  | X76 | Malignant neoplasm of the breast, in women |
|  | X77 | Other female genital neoplasms |
|  | X99 | Other female genital diseases |
| **Y** | Y77 | Malignant neoplasms of the prostate |
|  | Y78 | Other malignant neoplasms of male genital breast |
|  | Y85 | Benign prostatic hypertrophy |

^1^ O’Halloran, J.; Miller, G.C.; Britt, H. Defining chronic conditions for primary care with ICPC-2. Fam. Pract. 2004, 21, 381–386. <https://doi.org/10.1093/fampra/cmh407>.

^2^ International Classification of Primary Care, 2^nd^ Edition.

^3^ Not otherwise specified.

^4^ Human inmunodeficiency virus.

^5^ Peripheral vascular disease.

^6^ Chronic obstructive pulmonary disease.
